# Specificity of Thermal Destruction of Nonwoven Mixture Systems Based on Bast and Viscose Fibers

**DOI:** 10.3390/polym17091223

**Published:** 2025-04-29

**Authors:** Altynay S. Kalauova, Ekaterina E. Palchikova, Igor S. Makarov, Georgiy A. Shandryuk, Amangeldi I. Abilkhairov, Danagul Zh. Kalimanova, Meirbek Zh. Naukenov, Gulbarshin K. Shambilova, Egor M. Novikov, Junlong Song, Alexander G. Smyslov

**Affiliations:** 1 Department of Chemistry and Chemical Technology, Kh. Dosmukhamedov Atyrau University, Studenchesky Ave. 1, Atyrau 060011, Kazakhstan; skalauova@mail.ru (A.S.K.); dana80_04@mail.ru (D.Z.K.); shambilova_gulba@mail.ru (G.K.S.); 2 A.V. Topchiev Institute of Petrochemical Synthesis, Russian Academy of Sciences, 29 Leninsky Prospect, 119991 Moscow, Russia; gosha@ips.ac.ru (G.A.S.); alexsmislov@mail.ru (A.G.S.); 3 Institute of Petrochemical Engineering and Ecology Named After N.K. Nadirov, Atyrau Oil and Gas University Named After S. Utebayev, M. Baimukhanov Street 45A, Atyrau 060027, Kazakhstan; a.abilkhayrov@mail.ru; 4 LLP «Kazakhstan Petrochemical Industries Inc.», Atyrau-Dossor Highway Building 295, Atyrau 060000, Kazakhstan; meirbekk@mail.ru; 5 Department of Chemistry, New Mexico Highlands University, Las Vegas, NM 87701, USA; enovikov@live.nmhu.edu; 6 International Innovation Center for Forest Chemicals and Materials, Nanjing Forestry University, Nanjing 210037, China; junlong.song@njfu.edu.cn

**Keywords:** viscose, flax, hemp, cellulose fibers, nonwoven materials, pyrolysis, carbon yield, activation energy, Kissinger method

## Abstract

The research investigates the thermal behavior of mixed systems based on natural and artificial cellulose fibers used as precursors for carbon nonwoven materials. Flax and hemp fibers were employed as natural components; they were first chemically treated to remove impurities and enriched with alpha-cellulose. The structure, chemical composition, and mechanical properties of both natural and viscose fibers were studied. It was shown that fiber properties depend on the fiber production process history; natural fibers are characterized by a high content of impurities and exhibit high strength characteristics, whereas viscose fibers have greater deformation properties. The thermal behavior of blended compositions was investigated using TGA and DSC methods across a wide range of component ratios. Carbon yield values at 1000 °C were found to be lower for blended systems containing 10–40% by weight of bast fibers, with carbon yield increasing as the quantity of natural fibers increased. Thus, the composition of the cellulose composite affects carbon yield and thermal processes in the system. Using the Kissinger method, data were obtained on the value of the activation energy of thermal decomposition for various cellulose and composite systems. It was found that natural fiber systems have three-times higher activation energy than viscose fiber systems, indicating their greater thermal stability. Blends of natural and artificial fibers combine the benefits of both precursors, enabling the deliberate regulation of thermal behavior and carbon material yield. This approach opens up prospects for the creation of functional carbon materials used in various high-tech areas, including thermal insulation.

## 1. Introduction

Cellulose is one of the most abundant organic compounds on Earth and is of great importance both in nature and in industry [1]. The primary sources of plant cellulose are wood, cotton, flax, hemp, bamboo, and algae. Depending on the type, the cellulose percentage in wood ranges from 40 to 50%. In addition to cellulose, wood includes two additional components: lignin (20–30%) and hemicellulose (20–35%), which are mostly removed during cooking and complicate the production process [2]. Cotton is the purest natural source of cellulose (90–95%) and has a number of unique sorption and mechanical properties, such as high hygroscopicity, air permeability, pollution resistance, antiseptic properties, and high strength and wear resistance, due to which, cotton cellulose is used in the textile and medical industries (for example, for the production of gauze and bandages) [3]. Some seaweeds, such as red and brown algae, contain cellulose, albeit in minute amounts. Such sources are typically used in specialized applications, such as biotechnology.

Flax is one of the oldest cultivated plants and produces long, strong fibers. Flax fibers include 70–85% cellulose, up to 20% hemicellulose and pectin, and less than 5% lignin [4,5]. Growing and processing flax use less water and chemicals than cotton. Flax is utilized in the production of fabrics because of its strength, sorption, hypoallergenic characteristics, and air permeability [6].

Seed husks, grain shells, sawdust, and other biomass that were previously considered waste are now regarded as promising sources of cellulose due to their high yields and high fiber content, which can be used to make paper, textiles, biodegradable composites, and other eco-friendly materials. This includes industrial hemp [7]. Hemp grows quickly, absorbs a lot of CO_2_, does not degrade the soil, and can be farmed without chemicals [8]. Industrial hemp’s tolerance for unfavorable environments allows it to yield up to three harvests per year [9]. Hemp fibers comprise 65–75% cellulose, 15–20% hemicellulose, and 5–10% lignin [7,8,9,10,11].

Like flax, hemp stalks undergo a maceration procedure that includes soaking in water or exposing them to natural moisture (dew retting) in order to enzymatically break down the bonds that bind cellulose to other substances like lignin and hemicellulose [12]. Hemp stalks are either dew-retted (left in the field in a moist environment) or water-retted (submerged in water). The water activates microorganisms producing enzymes to break down the pectin molecules linking the fibers. Temperature, soil microbes, and natural precipitation all have an impact on the dew retting process [13,14]. Following maceration, the stalks are dried, then crushed and scutched to extract the fibers from the shives. This process involves mechanically extracting the primary and secondary fibers. In order to produce a fibrous phase that is enriched in cellulose, the maceration method uses less energy and chemicals. After maceration, bast fibers are chemically cooked if needed to produce fibrous cellulose with a larger percentage of alpha fraction. The fiber cellulose that is produced is used as a raw material to make textiles and paper [15]. Flax, hemp, and grains are becoming more popular as wood remains the main source of cellulose, since they are highly renewable, have low environmental impact, and can be used to produce environmentally friendly materials [15]. High-cellulose plant fibers like flax and hemp pulp are beneficial for use in construction, the textile industry, and in the creation of biodegradable materials. High strength, resilience to wear, and environmental friendliness are some of their special qualities [16].

Biodegradable nonwovens made of cellulose, starch, and other natural polymers are becoming increasingly popular among the materials [17]. They minimize environmental damage and cut down on the amount of plastic waste by replacing conventional synthetic counterparts. Flat structures made of fibers, threads, or other components joined mechanically, chemically, thermally, or in combination without the use of conventional knitting or weaving techniques are known as nonwoven materials [18]. Textiles, medicine, construction, packaging, and transportation are just a few of the many industries that employ them [19].

Nonwovens made of cellulose show promise as a substitute for synthetic materials in a number of industries. To fully replace synthetics, however, more advancements are required to increase their resilience to wear and water while also lowering production costs. Regenerated cellulose fibers like viscose, flax, and hemp are the primary sources of cellulose fibers for nonwoven fabrics. Viscose is now the most popular material for making cellulose nonwovens. Because of its softness, gloss, and superior hygroscopicity, viscose—one of the earliest artificial fibers made from wood—is used extensively in the textile industry [20,21]. However, the manufacturing method for viscose fibers has some drawbacks, such as the use of hazardous chemicals and significant energy and water consumption [22,23]. It uses carbon disulfide (CS_2_), a volatile and poisonous chemical that is bad for the environment and workers’ health. Sulfur-containing substances and industrial waste (such as sulfuric acid) contaminate water [24].

Cellulose fibers are joined mechanically (by needle-punching and hydroentangling), thermally (by thermal bonding), or chemically (by impregnation with binders) to form nonwoven materials [25]. One of the most popular techniques for creating nonwoven materials from natural fibers is needle-punching [26]. The main idea behind this technique is to use special needles to secure the fibrous web that is created (from monofilament raw materials or a mixture of different fibers). The needles stretch (entangle) the fibers in the transverse direction as they pass through the web, resulting in a nonwoven web that allows for the production of strong, long-lasting materials without the use of chemical binders. The process enables the creation of materials with a range of densities, thicknesses, and textures that are appropriate for a number of uses. It also enables the blending of fibers from various materials without the use of chemicals, if necessary [27]. The procedure uses comparatively little energy and is automated.

Using a blend of cellulose from multiple sources as a material for nonwoven products might be one way to reduce the environmental effect [28]. By incorporating natural fiber from hemp or flax into the nonwoven material, the percentage of viscose fiber can be decreased [29]. The production of natural fibers involves lower emissions, particularly carbon dioxide, than that of viscose fibers. In [30], the prospect of producing nonwoven textiles only from natural fibers was examined. It has been demonstrated that using flax and hemp fibers up to 42 mm in length prevents the production of flawless (monolithic) felt. Since using long flax fibers raises the cost of the final product significantly, it is not practicable. Artificial fibers that are at least 60 mm long and sufficiently crimped are needed to reinforce felt made of natural fibers. When cellulose fibers are carbonized, the carbon output can reach 44.4%, making cellulose a common precursor for carbon materials [31]. Because of their high carbon content, structural features, and potential to produce materials with specific qualities, nonwoven cellulose materials are a suitable precursor for the creation of carbon webs [32]. A cellulose-derived carbon web is utilized in composite materials for a variety of industries, batteries, supercapacitors, and filtration systems.

The attributes of the nonwoven fabric made from flax or hemp will vary when viscose fiber is changed entirely or in part. This will modify the fabric’s thermal behavior at high temperatures, potentially affecting the carbon fabric’s qualities. Using thermogravimetric analysis (TGA) at four different heating rates, the behavior of thermal decomposition (pyrolysis) of different plant fibers was investigated in [33]. The kinetics of the cellulose pyrolysis reaction were then described using techniques like the Friedman–Friedman, Flynn-Wall–Ozawa, and Kissinger methods [34,35,36]. Since pyrolysis is a very complex process, the mechanisms governing these breakdown processes remain unclear, even though the use of such procedures is widely recognized in the literature. It typically undergoes a sequence of reactions and is influenced by a number of variables, including temperature, heating rate, particle size and shape, catalyst presence, and initial biomass content [37,38,39,40]. According to [41,42], four stages of biomass heating can be identified on thermograms: moisture release, hemicellulose breakdown, cellulose breakdown, and lignin breakdown. According to the authors, the thermal effects of the three primary components might be superimposed during the pyrolysis of the biomass [43,44]. One of the most important factors in the material’s thermolysis is its structure [45].

Despite the large number of works on the thermal decomposition of pure cellulose and individual types of fibers, the thermal behavior of systems based on viscose and bast fibers has not yet been described in the literature and is not trivial. Thus, ref. [46] used a modified global first-order kinetic approach to estimate the activation energy of the pyrolysis of cotton and mercerized cotton, and refs. [47,48] analyzed the kinetics of the total mass of the biomass and its components. A number of works have been devoted to TGA/DSC of individual bast fibers (flax and hemp) or viscose [49,50,51]; however, a systematic research of binary mixtures of cellulose I and cellulose II in a wide range of concentrations and kinetic analysis has not yet been carried out, reducing the use of viscose fibers made using an environmentally dangerous process and making sensible use of natural fiber waste enabling by substituting natural fibers for artificial ones. We hypothesized that the proportion of viscose to natural fibers will be crucial in determining how much carbon residue is formed when the system is processed at high temperatures. The work’s purpose was to explore the thermal behavior of blended systems based on viscose and natural fibers across the entire concentration range, as nonwoven cellulose materials are a possible precursor for carbon nonwoven materials.

## 2. Materials and Methods

According to the patent [52], flax and hemp fibers were obtained in the form of tow with an average length of up to 25 mm (linear density 1.2 tex) (LenOm LLC, Kalachinsk, Russia). The company “Vostokhimvolokno” (Staraya Kupavna, Russia) provided the viscose fibers, which had a linear density of three deniers. Flax, hemp, and viscose fibers ranged in average diameter from 15 to 25 µm.

Using an inductively coupled plasma emission spectrometer (ICPE-9000, Shimadzu, Kyoto, Japan), the elemental chemical composition of the fibers was determined.

The alpha-cellulose content (insoluble fraction in 17.5% aqueous alkali solution) was re-estimated for all fibers [53]. Using a technique that involved treating the cellulose with 17.5% NaOH solution for 45 min, washing it with 9.5% NaOH solution and water, and then drying it, the amount of alpha-cellulose in the samples was ascertained. Between three and five trials were conducted.

The resin and fat content were estimated [54]. Measurements were conducted using dichloromethane (methylene chloride, CH_2_Cl_2_) stabilized with 0.08% ethanol. The method’s main steps involve extract cellulose using methylene chloride, then evaporate the extract, dry it, and weigh the non-volatile residue.

Following drying at (100 ± 5) °C, the mass change of the sample was used to calculate the moisture content in the fibers [55].

By measuring the inherent viscosity of solutions in cadoxene (cadmium oxide in ethylenediamine), the degree of polymerization of cellulose was ascertained [56]. An Ubbelohde capillary viscometer was used to evaluate the inherent viscosity of diluted cellulose solutions in cadoxene at 30 °C. The Mark–Kuhn–Houwink equation was used to determine the average degree of polymerization; the coefficient K_m_ and the exponent a were 0.71 and 0.93, respectively. Consequently, the average cellulose DP value was established. For every sample, the experiment was run at least three times, and the average inherent viscosity value was determined.

The structure of the materials was studied using X-ray diffraction. For X-ray diffractometry, we used a “Rigaku Rotaflex RU-200” (Rigaku Corporation, Tokyo, Japan) setup equipped with a rotating copper anode (linear focus 0.5 mm × 10 mm, source operating mode 50 kV–100 mA, wavelength of characteristic CuKα radiation λ = 1.542 Å, and secondary graphite monochromator); horizontal goniometer D-Max/B; and scintillation detector. X-ray imaging was performed in reflection geometry according to the Bragg–Brentano scheme in a continuous θ–2θ scanning mode in the angular range of 5–40° at a rate of 2 deg/min and a scanning step of 0.04°. The measurements were performed at room temperature.

Analysis of the O’Connor crystallinity index (I_1430_/I_895_) indicates that hemp fibers exhibit the highest degree of crystallinity, with an intensity ratio of 0.86, while viscose fibers show the lowest ratio at 0.53. According to the X-ray diffraction data, the degree of crystallinity is highest for hemp and lowest for regenerated cellulose, and the crystallite sizes were 40 Å (flax), 42 Å (hemp), and 35 Å (viscose) [57,58].

To evaluate the mechanical properties of flax, hemp, and viscose fibers, an Instron 1122 tensile testing machine (Instron, Norwood, MA, USA) was used with a base of 10 ± 0.01 mm, a stretching speed of 1 mm/min, and a temperature of 20 ± 2 °C [59]. Each group included 20 tests. Flax has a diameter of 16.2 ± 2 μm, tensile strength of 416 ± 90 MPa, elastic modulus of 7.1 ± 1.8 GPa, and elongation of 4.6 ± 1.3%. Hemp has a diameter of 90 ± 32 μm, demonstrates a higher strength of 630 ± 87 MPa, and has an elastic modulus of 13.5 ± 2.9 GPa, but its elongation is lower at 3.1 ± 0.8%. Viscose is characterized by a diameter of 19.7 ± 1.5 μm, strength of 352 ± 41 MPa, elastic modulus of 6.0 ± 0.9 GPa, and significantly higher elongation of 23.8 ± 2.9%.

Thermal behavior of the fibers was studied by TGA on a TGA/DSC1 Mettler Toledo thermal analysis device (Greifensee, Switzerland). Measurements were performed in 150 μL aluminum oxide crucibles in the temperature range from 30 to 1000 °C with a heating rate of 5, 10, and 20 K/min. The inert gas (argon) flow rate was 100 cm^3^/min. Each TGA curve was obtained 3–5 times to check the reproducibility of the data.

Kinetic studies were carried out using the Kissinger method [60]. The basic equation he used is derived from the Arrhenius equation and the reaction rate equation. In general, it has the form

(1) $$\ln\left(\frac{\beta }{T_{p}^{2}}\right)=-\frac{E_{a}}{RT_{p}}+const,$$

where *β*—the heating rate (°C/min or K/min), T_p_—the temperature at which the maximum reaction rate is achieved (peak on the DSC or TGA curve), R—the universal gas constant, and E_a_—the activation energy.

The method consists of conducting a series of experiments with different heating rates: *β*_1_, *β*_2_, …, *β_n_*. Then, the peak temperatures T_p_ are recorded for each heating rate. Afterwards, a graph of the dependence of ln(β/T_p_^2^) on 1/T_p_ is constructed, and the slope of the resulting line is determined, which is equal to −E_a_/R.

## 3. Results

Given their origin and structure, natural and viscose fibers have different mechanical properties. Bast fibers form naturally, giving them distinct features, whereas viscose fibers are created purposefully, allowing their attributes to be regulated. Viscose fibers are more flexible and stretchable, while natural fibers, especially hemp, have high strength and rigidity. Artificial fibers differ from natural ones not only in structure and mechanical characteristics but also in a number of other characteristics. The concentration of alpha cellulose, resins, and fats; the amount of adsorbed moisture; and the degree of polymerization were all measured for each fiber sample in compliance with the criteria; the findings are shown in Table 1.

During pulping, low-molecular substances are removed due to their solubility in an alkaline medium, and fatty acids, esters, and waxes are saponified and hydrolyzed. In the later stages of processing, a decrease in pH can cause precipitation of these compounds on the fibers [61]. During the spinning of viscose fibers, heteropolymers are precipitated on their surface during coagulation [62]. The precipitated compounds degrade the quality of the fibers, reducing the interfiber interactions and mechanical properties due to their hydrophobicity.

Viscose fibers are spun from dissolving pulp, which has strict requirements regarding the content of organic and inorganic impurities. In natural fibers, on the other hand, various cellulose satellites may be present, and their quantity is determined by the place of growth and processing conditions. The content of inorganic impurities in the fibers used to form nonwoven materials is presented in Table 2.

The results of the elemental analysis show that the metal content depends on the type of fiber. Natural fibers are characterized by a high content of calcium and magnesium, while viscose fibers are dominated by sodium and zinc, which is due to the technological features of their production. These differences affect the properties of the fibers. For example, an iron content above 8 ppm worsens thermal stability, and sodium in cellulose should not exceed 85 ppm. Particular attention should be paid to magnesium; its content in natural fibers is 10 times higher than in viscose fibers, where it corresponds to the recommended level (up to 12 ppm). It is important to note that the chemical composition of the fibers [63,64] and crystallinity/crystal size [65,66] significantly affects the mechanical and functional properties of the resulting materials.

Since viscose and hemp (flax) fibers are utilized to make nonwovens that may be employed not only as filters, insulating materials, and so on but also as precursors to carbon nonwovens, the thermal behavior of such fibers was investigated as a first step.

**Figure 1:**
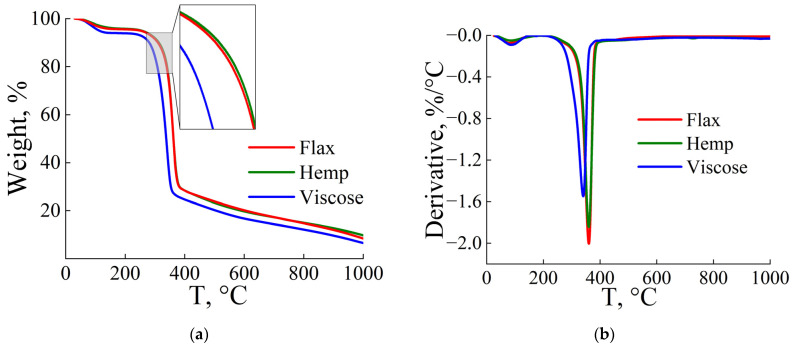
TGA (**a**) and DTG (**b**) curves of flax, hemp, and viscose fibers.

Figure 1 shows the TGA and DTG thermograms for individual fibers, which are similar in nature. The curves can be divided into three main sections. In the first section, up to 30–180 °C, 4–6% of the adsorbed water mass is removed without signs of thermal destruction. In the second section, starting at 200 °C, there is a sharp loss of mass for all samples, with two competing reactions occurring: dehydration and depolymerization [67]. For viscose fibers, the destruction processes begin at lower temperatures compared to natural fibers. In the last section, in the range from 400 to 1000 °C, a gradual decrease in mass is observed, caused by the loss of oxygen and hydrogen.

To study the thermal behavior of the compositions, the required amount of flax or hemp fibers was added to the volume of viscose fibers, then the mixtures were mechanically mixed. As a result, more or less homogeneous systems of fibers of different nature were obtained with a random distribution of native cellulose I (natural fibers) in the matrix of cellulose II (viscose staple). The results of the thermal study of mixed compositions of different concentrations are presented in Table 3 and Table 4.

Table 3 shows how the pyrolysis onset temperature, carbon yield, and peak thermal decomposition rate change, depending on the composition of the samples. For example, flax and viscose have similar carbon yield values, but these values can change when the ratio of natural and manmade fibers in the mixture changes. Similar thermal properties of compositions containing hemp were also obtained and studied (Table 4).

**Figure 2:**
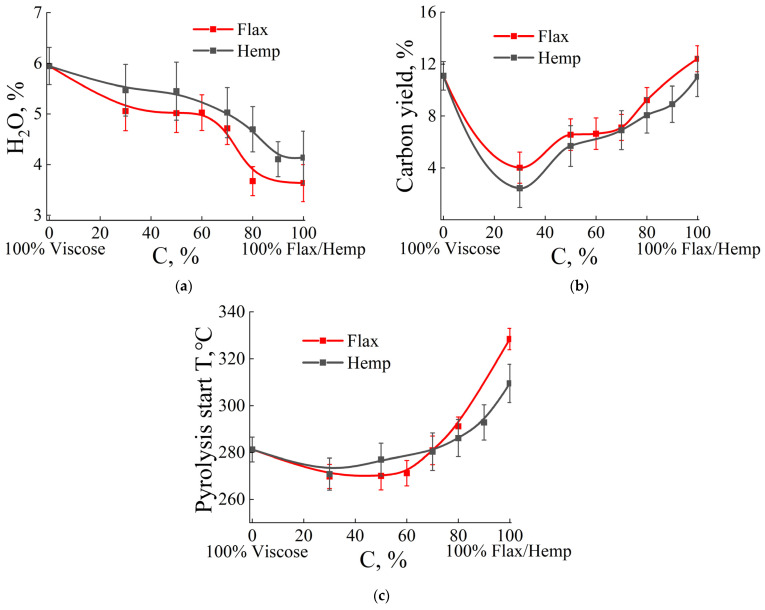
TGA and DSC results. Concentration dependence of the water content (**a**), carbon yield at a temperature of 1000 °C (**b**), and the temperature of the onset of pyrolysis on the content of flax and hemp fibers in the mixed compositions (**c**).

**Figure 3:**
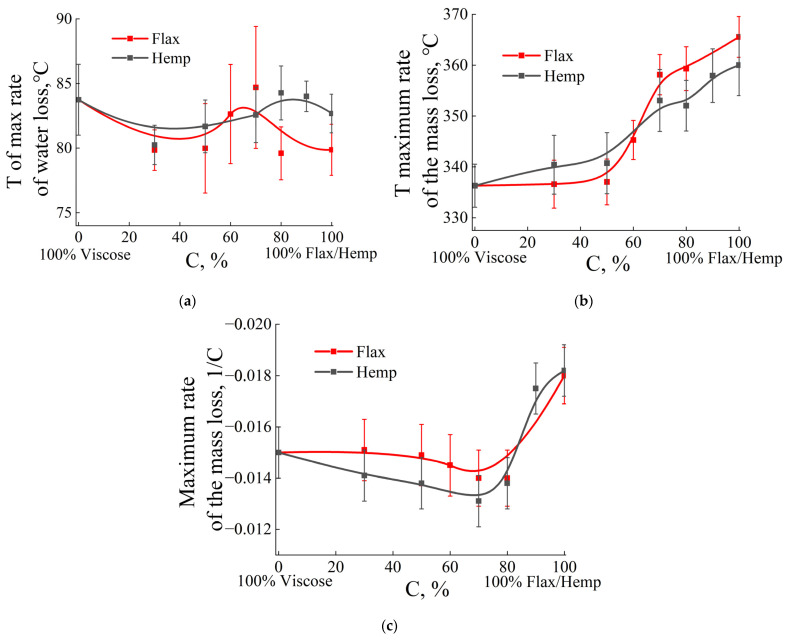
DTG results. Concentration dependence of the water loss rate (**a**), T of the maximum mass loss rate (**b**), and maximum rate of the mass loss (**c**) of fiber-mixed compositions.

The thermal analysis data for systems with different ratios of artificial and natural fibers are presented as concentration dependencies for visualization (Figure 2 and Figure 3).

The thermal behavior of viscose fibers starts to alter when natural fibers are added. Figure 2a illustrates how the percentage ratio of the constituents affects the water content in samples of viscose and natural (flax and hemp) fiber mixtures. Viscose fibers have the most water (about 6%), while flax and hemp fibers contain less water (3.5–4%). For up to 40–60% natural fiber concentration in mixtures, slight variations in the moisture content are seen when natural fibers are added to viscose fibers.

In mixtures with a predominance of natural fibers, the total water content decreases. Structural differences between flax and hemp fibers, as well as different chemical composition, affect the values of the total content of adsorbed moisture in the system. For mixtures with hemp fibers, higher values of adsorbed moisture are observed compared to systems with flax fibers of the same concentration.

The concentration dependence presented in Figure 2b allows us to analyze the effect of replacing viscose fibers with natural ones on the amount of carbon yield. Adding a tiny amount of natural fibers (up to 30%) to viscose results in a significant drop in carbon yield. However, increasing their concentration in the mixture leads to the creation of carbon material with a higher mass. At the same time, changes in the amount of carbon yield are almost completely independent of the type of natural fibers used, with the nature of the dependence dictated solely by the concentration of additional flax or hemp fibers.

The temperature of the onset of pyrolysis depends on the structure of the fibers and the presence of impurities in them. Bast fibers have a higher degree of ordering than viscose fibers. Therefore, with an increase in the proportion of such fibers in the system, an increase in the values of the temperature of the onset of pyrolysis is observed. Flax fibers have a lower content of calcium and magnesium compared to hemp. Hence, one can expect a greater catalytic activity of impurities, which is accompanied by a decrease in the temperature of the onset of pyrolysis [68,69,70]. When the content of bast fibers is higher than 70%, the observed difference in the temperatures of the onset of pyrolysis tends to be the maximum.

Viscose fibers contain small amounts of Ca, Mg, and Fe compared to hemp and flax [68,71,72,73]. However, the sodium content is almost an order of magnitude higher compared to bast fibers. It is possible that low-purity water was used in the process of spinning viscose fibers. Interestingly, sodium can also affect the thermal behavior of cellulose fibers and, as shown in [68], can promote accelerated decomposition, i.e., the thermal decomposition temperature of the polymer decreases. Thus, the introduction of flax and hemp into viscose fibers affects the temperature at which pyrolysis begins, shifting it to higher temperatures.

Figure 2c shows the dependence of the pyrolysis onset temperature on the flax and hemp fiber content in the blended composites. At the beginning, with a low flax and hemp percentage, the decomposition temperature remains nearly constant. As their concentration rises, a gradual temperature rise is noted, which becomes notably apparent at 80%. According to the results of the quantitative chemical analysis (Table 2), hemp has more calcium and magnesium concentration than flax, indicating better catalytic activity and a lower pyrolysis initiation temperature [68,69,70]. This is consistent with the graph, which shows that the pyrolysis initiation temperature for hemp remains lower. At the same time, viscose has significantly fewer catalytic elements (Ca, Mg, and Fe) than hemp and flax [68,71,72,73]. However, it contains an order of magnitude more sodium, which can contribute to accelerated decomposition. Sodium acts as a catalyst for cellulose pyrolysis, reducing its decomposition temperature. Thus, the introduction of these natural fibers into viscose increases the thermal stability of the material, with flax having a more pronounced effect on this indicator compared to hemp.

From the point of view of developing high-temperature heat-protective materials, natural fibers have greater heat resistance than viscose fibers. The rate of water mass loss is similar across compositions and water concentrations in fibers (Figure 3a), with a range of 79–85 °C. This indicates that adsorbed moisture is removed from the fiber surface. That is, the first stage of heating (up to 30–180 °C) occurs under the same conditions. This means that water adsorbed on the surface of the fibers is removed by similar mechanisms, regardless of the type and proportion of fibers in the system. The next section of the thermogram corresponds to the pyrolysis of cellulose. The maximum rate of mass loss in viscose is 25% lower compared to flax and hemp fibers (Figure 3b).

At a low natural fiber content, the rate of decomposition remains relatively low, with flax and hemp samples behaving similarly. With an increase in the proportion of natural fibers (flax or hemp), the maximum rate of mass loss increases, which is consistent with the previous graphs, where viscose “softens” the pyrolysis process. After exceeding the threshold of about 60% natural fibers, a sharp increase in the maximum rate is seen, with this jump being slightly more pronounced for flax than for hemp. Thus, Figure 3c illustrates the same trend: viscose at low proportions of flax or hemp reduces the rate of decomposition, but with a predominance of natural fibers, the pyrolysis process accelerates significantly, with flax demonstrating a sharper increase than hemp.

Thus, the results of thermogravimetric analysis (TGA) show the influence of the fiber type and its concentration on the thermal characteristics of the composition. It is interesting to note that, in polymer composite systems, the concentration range of 40–60% is a boundary region, where it is difficult to predict the properties of the resulting composition [74,75], and a similar situation is observed in the presented mixed systems. Systems containing flax (hemp) and viscose in the concentration range of 40–60% exhibit unstable thermal behavior (“worrying region”). A careful approach is required to obtain carbon nonwoven materials based on precursors with such a ratio of components.

In natural cellulose tows, the fiber length does not exceed 25 mm, which does not allow obtaining a nonwoven fiber with a dense mesh of interlocks. During thermal treatment of such materials, the frequency of the mesh of such interlocks can decrease, which is associated with a decrease in the length and thickness of cellulose fibers. Therefore, the formation of precursor nonwoven materials based on tows is difficult. Their reinforcement requires the presence of long fibers, which are viscose fibers. Based on this, the optimal concentration of tows in the system is 70–80%, and viscose fibers no more than 30%.

When developing new cellulose-based materials, it is important to consider the conditions of their thermal decomposition, which is especially important for biocomposites, packaging materials, heat-resistant fibers, and textiles based on them. When thermally processing such products, it is necessary to take into account not only the temperatures of the onset of pyrolysis, etc. but also the activation energy of pyrolysis. This allows choosing the optimal temperature conditions for the carbonization of nonwoven and other precursors. The Kissinger method, due to its simplicity, has become widely used to determine activation energies [76]. The Kissinger mathematical model is applied to irreversible reactions occurring during heating at a constant rate and is based on dynamic thermal analysis, such as differential scanning calorimetry (DSC) or thermogravimetric analysis.

**Figure 4:**
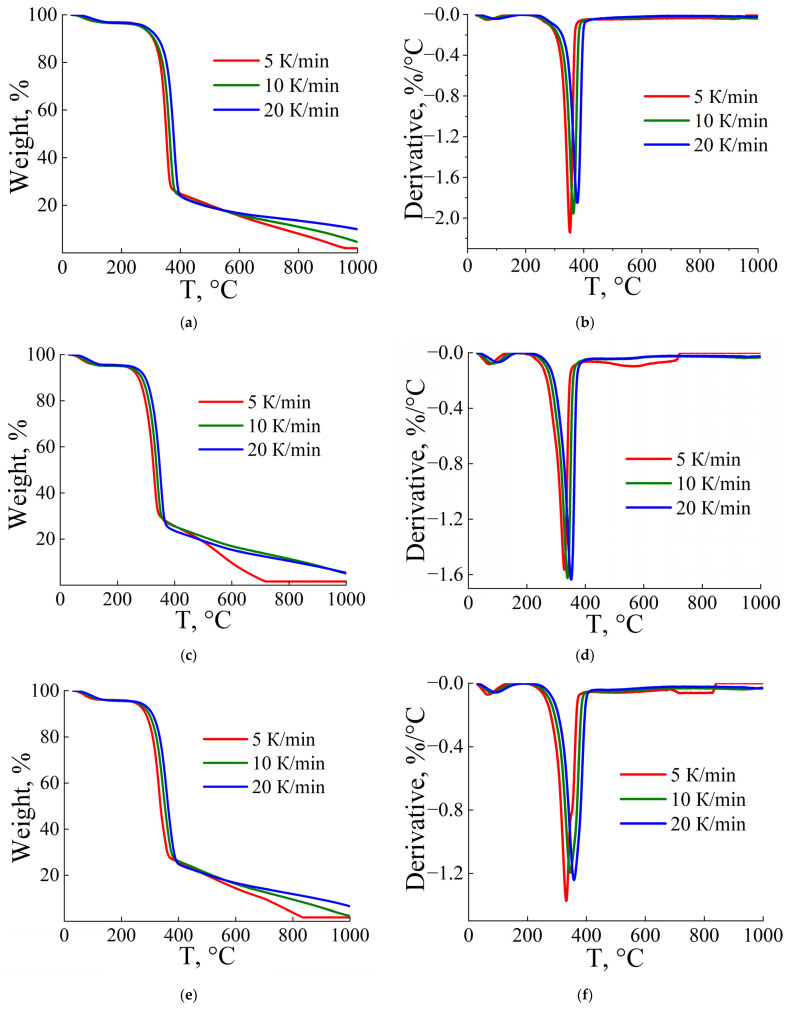
TGA (**a**,**c**,**e**) and DTG (**b**,**d**,**f**) curves of flax fibers (**a**,**b**), viscose (**c**,**d**), and a 50/50% wt. blend of flax and viscose fibers (**e**,**f**).

A series of experiments was carried out with different heating rates of 5, 10, and 20 K/min for two samples (viscose and flax) and their composition (50% viscose and 50% flax). Since the thermal behavior of flax and hemp fibers is similar (Figure 1), it can be assumed that the kinetic parameters of their thermal decomposition will be the same. Figure 4 shows the TGA and DTG curves of flax, viscose, and their mixture of fibers. The temperature range of pyrolysis and the temperatures of the peaks T_p_ for each heating rate, determined from the first derivative of the TGA curves, were used to estimate the activation energy of thermal decomposition. According to [47], these values allow not only to estimate the energy required for the reaction but also the possible nature of the resulting product. The corresponding temperature values at which the maximum reaction rate is achieved (the peak on the DTG curve) are presented in Table 5.

As the heating rate (β) increases, the peak temperature (T_p_) increases for all materials. Flax shows the highest T_p_ values among all the samples, while viscose shows the lowest. This difference is due to the fact that flax consists of cellulose I, which has a higher level of crystallinity, unlike viscose, which is a regenerated cellulose with a lower degree of crystallinity, making it more prone to thermal decomposition at lower temperatures [27]. The composite fiber (50% flax/50% viscose) has intermediate T_p_ values.

**Figure 5:**
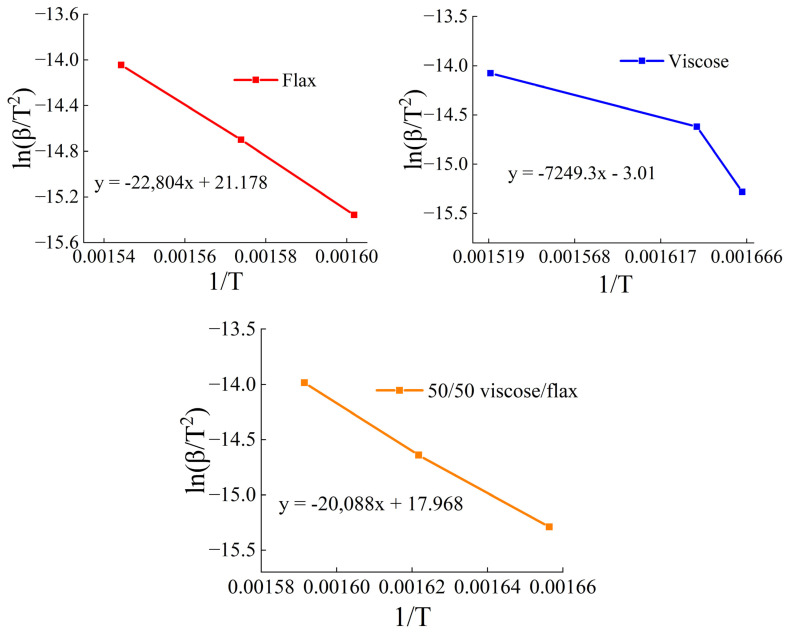
Dependence of ln(β/T_p_^2^) on 1/T_p_ for determining the activation energy of thermal decomposition.

Using the obtained data, a graph of the dependence of ln(β/T_p_^2^) on 1/T_p_ was then constructed, and the values of the activation energies E_a_ were determined from the slope of the line (Figure 5, Table 6).

The study [46] focused on the kinetics of the pyrolysis of cellulose fibers. Using a modified first-order model based on TGA data, the authors analyzed the pyrolysis of two types of fibers: natural cotton yarn and mercerized cotton. The technique allows extrapolating the data to a temperature approaching zero, which ensures the determination of the “internal” activation energy of pyrolysis. The obtained values are about 153 ± 2 kJ/mol for natural cotton yarn and 192 ± 7 kJ/mol for mercerized cotton, indicating the influence of differences in the chemical composition and structure of the fibers on their pyrolytic reactivity. Van de Velden’s studies show activation energy values of 54.1–86.4 kJ/mol for the general process of biomass pyrolysis [54]. The works [49,50,51,77,78,79] reported activation energies of cellulose pyrolysis in the range of 135–223 kJ/mol. It is noted that the obtained parameters depend on the measurement method used and the type of starting material. Thus, the values found for mixed systems based on viscose and natural fibers correlate with the values previously described in the literature for cellulose and fibers based on it.

The activation energy of the thermal decomposition of viscose can be increased by adding a material with higher ordering (crystallinity), such as flax. For a system containing 50% flax and 50% viscose fibers, the thermal behavior was studied, and the activation energy (E_a_ = 167.01 kJ/mol) was calculated, which is lower than the values for flax-based systems and higher than the values for viscose-based systems. Adding flax to viscose fibers changes the thermal behavior of the system with increasing activation energy values. By adjusting the concentration of flax in the system, it is possible to change E_a_, i.e., the system becomes more resistant to thermal decomposition and allows obtaining materials with the required structure (network of interlocks) and mechanical properties.

Another way to modify and stabilize the pyrolysis mechanism and, accordingly, increase the activation energy is to use suitable chemicals such as impregnations or fire retardants that accelerate and catalyze dehydration reactions in order to reduce weight loss, improve yields and properties of final products, and achieve higher efficiency [80]. For example, the work [81] studied the efficiency and effect of ammonium sulfate on the oxidative thermal stabilization of activated fiber based on viscose fiber. The fibers are pre-impregnated with an ammonium sulfate solution, which promotes their stabilization before carbonization and subsequent activation. As a result of the treatment, significant physicochemical transformations are observed: the thickness and linear density of the fibers decrease, the color changes (from white to black), the crystallinity of the cellulose decreases, and the transformation of the primary hydroxyl groups into carbonyl groups is recorded, which makes them suitable for the production of carbon fibers. A more environmentally friendly way, without the use of chemical reagents, is to add reinforcing fillers [82]. The introduction of nanocellulose fibers or graphene nanoparticles, which strengthen the structure of the material, or the addition of lignin or polysaccharides, which form a more stable network inside the viscose [83,84]. These studies provide a good understanding of the energy barriers of cellulose pyrolysis and confirm that the temperature and chemical properties of the starting materials play a major role in the thermal processing of carbon fibers.

The current study did not involve the use of pyrolysis catalysts and fire retardants. Therefore, in the future, it is of interest to study the thermal behavior of nonwovens based on viscose and natural fibers with their preliminary treatment with special compounds. Such treatment will increase the observed values of carbon yield and possibly affect the activation energy of pyrolysis.

## 4. Conclusions

Cellulosic nonwoven materials can be made from both artificial (viscose) and natural fibers (flax and hemp). The chemical composition, structure, and properties of these fibers are determined by their nature and the method of their production. Though they show less deformation characteristics, natural fibers are stronger than viscose. Although the fibers used have different structural and chemical characteristics, the viscose to fibrous flax (hemp) cellulose ratios control the thermal behavior of blended systems. The system’s total moisture content gradually decreases as the quantity of natural fibers increases. The rate of water loss, depending on the ratio of the components, undergoes minor changes. The addition of a small amount of bast fibers decreases the carbon yield, but at concentrations exceeding 60%, it increases dramatically. The rate of mass loss during pyrolysis, on the contrary, increases with an increase in the content of the bast fiber phase. The Kissinger method showed that flax fibers have the highest thermal stability (activation energy of 189.59 kJ/mol), and viscose has the lowest (60.27 kJ/mol). An equiconcentrated system (50% viscose and 50% flax) demonstrates average thermal stability (167.01 kJ/mol). The bast tow does not provide a dense mesh of adhesion due to its short length, which makes it difficult to obtain carbon material based on it. The optimal blend for nonwovens is 70–80% bast tow and 20–30% viscose, which supports the nonwoven due to its long length and the creation of interlocks between the fibers. In order to obtain high-quality precursors and maximum carbon output, it is necessary to replace viscose fibers with natural ones. The correct choice of pyrolysis catalysts and fire retardants will allow achieving higher carbon output values in the future.

## Figures and Tables

**Table 1:** Content of resins and fats, alpha fraction of cellulose, moisture, and degree of polymerization (average values) for viscose and flax fibers.

Fiber Type	α—Fraction, %	H_2_O, %	Degree of Polymerization	Fats and Resins, %
Flax	97.4	5	>1000	0.48
Hemp	98	4.2	>1000	0.43
Viscose	91	5.9	256	0.29

**Table 2:** Content of inorganic impurities in fibers.

Element	Content in Fibers, ppm		
	Flax	Viscose	Hemp
Al	228	44	116
Ca	899	105	2905
Fe	130	69	177
K	102	132	77
Mg	260	12	640
Na	614	2785	1637
Zn	13	38	23

**Table 3:** TGA data for flax fibers and mixtures with viscose.

Sample	Water, %	Pyrolysis Start Temperature, °C	Carbon Yield, %	T of Maximum Rate of Water Loss, °C	T Maximum Rate of the Mass Loss, °C	Maximum Rate of the Mass Loss, 1/°C
100% Flax	3.63 ± 0.365	328.41 ± 4.56	12.415 ± 1	79.87 ± 1.98	365.55 ± 4	−0.018 ± 0.0011
80% Flax/20% Viscose	3.673 ± 0.287	291.24 ± 3.94	9.27 ± 0.97	79.6 ± 2.05	359.32 ± 4.31	−0.014 ± 0.001
70% Flax/30% Viscose	4.716 ± 0.321	280.96 ± 6.1	7.12 ± 1	84.7 ± 4.72	358.13 ± 3.96	−0.014 ± 0.0011
60% Flax/40% Viscose	5.027 ± 0.353	271.19 ± 5.46	6.64 ± 1.21	82.64 ± 3.84	345.29 ± 3.84	−0.0145 ± 0.0012
50% Flax/50% Viscose	5.015 ± 0.379	270.03 ± 6	6.56 ± 1.21	79.99 ± 3.46	337.04 ± 4.5	−0.0149 ± 0.0012
30% Flax/70% Viscose	5.055 ± 0.385	269.78 ± 5.17	4.02 ± 1.2	79.85 ± 1.57	336.6 ± 4.71	−0.0151 ± 0.0012
100% Viscose	5.946 ± 0.368	281.3 ± 5.3	11.1 ± 1.1	83.75 ± 2.74	336.3 ± 4.23	0.015 ± 0.001

**Table 4:** TGA data for hemp fibers and mixtures with viscose.

Sample	Water, %	Pyrolysis Start Temperature, °C	Carbon Yield, %	T of Maximum Rate of Water Loss, °C	T Maximum Rate of the Mass Loss, °C	Maximum Rate of the Mass Loss, 1/°C
100% Hemp	4.139 ± 0.522	309.54 ± 8.17	11.03 ± 1.53	82.68 ± 1.49	360.01 ± 5.98	−0.0182 ± 0.001
90% Hemp/10% Viscose	4.107 ± 0.345	292.87 ± 7.55	8.91 ± 1.4	84 ± 1.18	357.95 ± 5.27	−0.0175 ± 0.001
80% Hemp/20% Viscose	4.698 ± 0.447	286.17 ± 7.93	8.06 ± 1.37	84.27 ± 2.09	352.04 ± 4.98	−0.0138 ± 0.001
70% Hemp/30% Viscose	5.027 ± 0.496	280.37 ± 8.01	6.91 ± 1.5	82.57 ± 2.14	353.06 ± 6.09	−0.0131 ± 0.001
50% Hemp/50% Viscose	5.452 ± 0.572	277 ± 7.03	5.69 ± 1.57	81.68 ± 2.04	340.73 ± 6.01	−0.0138 ± 0.001
30% Hemp/70% Viscose	5.469 ± 0.512	270.8 ± 6.87	2.44 ± 1.49	80.25 ± 1.52	340.43 ± 5.78	−0.0141 ± 0.001
100% Viscose	5.946 ± 0.368	281.3 ± 5.3	11.1 ± 1.1	83.75 ± 2.74	336.3 ± 4.23	−0.015 ± 0.001

**Table 5:** DTG data for flax, viscose, and the fiber mixture.

β, K/min	T_p_, °C		
	Flax	50% Flax/50%Viscose	Viscose
5	351.28	330.72	328.16
10	362.36	343.62	337.65
20	374.55	355.35	384.86

**Table 6:** Activation energies of thermal decomposition for flax, viscose, and the fiber mixture.

Sample	E_a_, kJ/mol
Flax	189.59
50% Viscose/50% Flax	167.01
Viscose	60.27

## Data Availability

The original contributions presented in the study are included in the article; further inquiries can be directed to the corresponding author/s.
